# Academic Aspirations

**DOI:** 10.31486/toj.26.5061

**Published:** 2026

**Authors:** Ronald G. Amedee

**Affiliations:** Director of Clinical School and Professor, The University of Queensland Medical School, Ochsner Clinical School, New Orleans, LA; Editor-in-Chief, *Ochsner Journal*

*The mediocre teacher tells. The good teacher explains. The superior teacher demonstrates. The great teacher inspires.*

–*William Arthur Ward*

The Summer 2026 issue of the *Ochsner Journal* contains two original research articles, eight case reports and clinical observations, one historical vignette, and a memorial to a fallen Ochsner Health physician-scientist, all of which serve as the principal elements for this edition.

The historical vignette details the surgery that likely saved pro golfer Ben Hogan's life after a devastating automobile accident in 1949. The surgeon was Dr Alton Ochsner.

The first original research article is from Vijayakumar (Ochsner radiology) and Prabhakaran, Nikitha S, and Rao (from Manipal, India) entitled “Comparative Distribution of High-Risk Human Papillomavirus (HPV) Genotypes in India vs World Health Organization Regions and Countries: Implications for HPV Vaccination.” This article is followed by “Development and Implementation of a Radiation Safety Screening Protocol for a Clinical Study Using Amyloid Positron Emission Tomography Imaging” authored by Fong, Martine, Heine, Joffe, Liu, Topper, Schmitt, Kuo, Redlich, and Inouye from Beth Israel Deaconess Medical Center/Harvard Medical School, Hofstra University/Norwell Health, the University of Rochester School of Medicine and Dentistry, City of Hope National Medical Center, the University of Florida, and Yale School of Medicine.

The case reports and clinical observations section begins with “Revision Anterior Cruciate Ligament Reconstruction in an Adult Twin With Meier-Gorlin Syndrome” authored by Suri, Mudiganty, Sclafani, Godshaw, and Montgomery from Ochsner orthopedics, and Vanegas-Contla from Hospital Excel in Tijuana, Mexico. Next is a submission from Linscheid, Willard, Schoofs, Few, and Jones from Ochsner orthopedics and emergency medicine entitled “Bilateral Trochleoplasty Procedures Following Bilateral Femoral Rotational Osteotomies for Bilateral Dejour Type D Trochlear Dysplasia.” Another submission from Ochsner orthopedics and emergency medicine faculty is “Type 1 Collagen/Poly-L-Lactic Acid Biocomposite Augmentation of a Nontraumatic Symptomatic Complete Rotator Cuff Tear” authored by Wilder, Willard, Schoofs, Bennett, Jacobs, Laurent, and Jones. This paper is followed by “Postcholecystectomy Biliary Tract Traumatic Neuroma Mimicking Cystic Duct Stump Malignancy” by Rai, Soni, Rao, Sureka, Saikrishna, Selvakumar, Varshney, and Agarwal from the All India Institute of Medical Sciences in Jodhpur, India. Another All India Institute of Medical Sciences submission, this one from Bhopal, India, is entitled “Spurious Plasma Adrenocorticotropic Hormone Level in a Patient With Adrenal Cushing Syndrome” authored by Chandran, Goyal, Mane, Gupta, and Choudhary. “Revumenib-Induced QTc Prolongation Leading to Multiple Episodes of Torsades de Pointes and Ventricular Arrhythmias” is by Keller, Hermanson, Tawwater, Schuller, St. Pierre, Naina, and Alzghari from Texas Health Harris Methodist and Texas Oncology in Fort Worth, Texas. Authors Thammakosol, Vathesatogkit, Leelasithorn, and Sriphrapradang from Ramathibodi Hospital, Mahidol University, Bangkok, Thailand, submitted “Primary Ovarian Neuroendocrine Neoplasm With Carcinoid Syndrome and Carcinoid Heart Disease.” The final case is “Recurrent Seizure-Like Events in a Toddler With BMPR2-Related Pulmonary Arterial Hypertension” authored by Cagatay, Ahluwalia, and Luat from Children's Hospital of Michigan in Detroit.

Every physician in academic practice ultimately wishes to be fondly remembered by learners, colleagues, and patients. From a teaching perspective, the above quote from William A. Ward encapsulates the aspiration of academicians to not only tell, explain, and demonstrate to our learners but also to inspire them—our future physician colleagues. Sometimes, an individual enters our professional lives who has achieved mastership as a clinician, a researcher, and a medical teacher. Dr George Atkinson Pankey reached this level, and I strongly urge you to read the memorial dedicated to him in this issue. Ochsner Health was incredibly blessed by his presence, example, and accomplishments. He will be deeply missed.

